# The effects of substituting red and processed meat for mycoprotein on biomarkers of cardiovascular risk in healthy volunteers: an analysis of secondary endpoints from Mycomeat

**DOI:** 10.1007/s00394-023-03238-1

**Published:** 2023-08-25

**Authors:** Dominic N. Farsi, Jose Lara Gallegos, Tim J. A. Finnigan, William Cheung, Jose Munoz Munoz, Daniel M. Commane

**Affiliations:** 1 Applied and Health Sciences, University of Northumbria, Sutherland Building, Ellison Place, Newcastle upon Tyne, NE1 8ST UK; 2 NUTRAN, Northumbria University, Newcastle upon Tyne, UK; 3 Marlow Foods, Stokesley, TS9 7AB UK

**Keywords:** Meat replacement, Meat alternatives, Plant-based, Mycoprotein, Fungal glucan, Fibre, Cardiometabolic

## Abstract

**Purpose:**

Mycoprotein is a relatively novel food source produced from the biomass of *Fusarium venenatum*. It has previously been shown to improve CVD risk markers in intervention trials when it is compared against total meat. It has not hitherto been assessed specifically for benefits relative to red and processed meat.

**Methods:**

We leveraged samples from Mycomeat, an investigator-blind randomised crossover controlled trial in metabolically healthy male adults (n = 20), randomised to consume 240 g/day of red and processed meat for 14 days followed by mycoprotein, or vice versa. Blood biochemical indices were a priori defined secondary endpoints.

**Results:**

Mycoprotein consumption led to a 6.74% reduction in total cholesterol (*P* = 0.02) and 12.3% reduction in LDL cholesterol (*P* = 0.02) from baseline values. Change in fasted triglycerides was not significantly different between diets (+ 0.19 ± 0.11 mmol/l with mycoprotein, *P* = 0.09). There was a small but significant reduction in waist circumference for mycoprotein relative to meat (− 0.95 ± 0.42 cm, *P* = 0.04). Following the mycoprotein diet, mean systolic (− 2.41 ± 1.89 mmHg, *P* = 0.23) and diastolic blood pressure (− 0.80 ± 1.23 mmHg, *P* = 0.43) were reduced from baseline. There were no statistically significant effects of the intervention on urinary sodium, nitrite or TMAO; while urinary potassium (+ 126.12 ± 50.30 mmol/l, *P* = 0.02) and nitrate (+ 2.12 ± 0.90 mmol/l, *P* = 0.04) were both significantly higher with mycoprotein relative to meat. The study population comprised metabolically healthy adults, therefore, changes in plasma lipids had little effect on cardiovascular risk scores (− 0.34% FRS for mycoprotein *P* = 0.24).

**Conclusions:**

These results confirm potential cardiovascular benefits when displacing red and processed meat with mycoprotein in the diet. Longer trials in higher risk study populations are needed to fully elucidate suggested benefits for blood pressure and body composition.

ClinicalTrials.gov Identifier: NCT03944421.

**Supplementary Information:**

The online version contains supplementary material available at 10.1007/s00394-023-03238-1.

## Introduction

Observational studies suggest that red and processed meat consumption is associated with an increased risk of cardiovascular disease (CVD) [1–5]. In contrast, adherence to plant-based dietary patterns appears to confer a cardioprotective effect [6, 7]. Notably, findings from one prospective cohort study suggest a 10% increase in energy intake from animal protein translates into an 8% increased risk of CVD mortality, while a 3% increase in energy from plant protein reduces risk by 12% [8]. These observations are reciprocated by findings from randomised controlled trials that report favourable effects in CVD risk markers when meat consumption is curtailed [9–12], including lower low-density lipoprotein (LDL) cholesterol and apolipoprotein B levels [13]; as well as reduced urinary excretion of microbially produced trimethylamine N-oxide (TMAO) [14].

Public health approaches have had limited success in inducing meaningful population level transitions to plant-based eating, due to culinary traditions, taste preferences, and social and cultural norms [15–18]. Meat alternatives—i.e., vegetarian and/or vegan foods designed to mimic meat, yet devoid of animal meat (vegetarian) or any animal derivative (vegan)—offer an effective approach to curtail meat consumption without drastically altering meal patterns and dietary habits [19, 20]. Mycoprotein is a sustainable protein derived from the continuous cultivation of *Fusarium venanatum*, which is high in protein and fibre, while low in fat [21–23]. With processing, it can resemble the texture and flavour of meats.

Previous randomised controlled trials have found replacing total meat with mycoprotein based foods elicits reductions in total and LDL cholesterol. These trials report different responses in high density lipoprotein (HDL), and this may be due to differences in the study population demographics and baseline cardiometabolic disease risk status [24–26]. It is important therefore to better characterise the effect of displacing meat with mycoprotein on CVD risk markers in wider study populations. In addition, the comparative influence on CVD markers of mycoprotein with that of red and processed meat have not been investigated. Given that epidemiological evidence suggests a greater risk of CVD from red and processed meat versus other animal protein sources [27], such investigations would be informative to nutritional science and public health.

Here, we present an analysis of secondary endpoints from the Mycomeat study. Mycomeat was a randomised crossover controlled dietary intervention trial comprising metabolically healthy males with faecal genotoxicity as the primary endpoint; a surrogate marker of colorectal cancer risk by evaluating the DNA damaging potential of foodstuff and dietary patterns within the colon [28]. The participants consumed a variety of red and processed meat products or the weight equivalent of mycoprotein based products over 2 week diet phases. Blood biochemical parameters related to CVD risk were included as a priori defined secondary endpoints.

## Methods

### Study setting and participants

Mycomeat has been described in detail elsewhere [28] (ClinicalTrials.gov Identifier: NCT03944421). Procedures were followed in accordance with the declaration of Helsinki and the study was approved by the Northumbria University Ethics Committee (reference number 15274). The participants involved in the study provided written informed consent.

Study participants were metabolically healthy male adults recruited from the North East of England, UK, via poster advertisement and using a database of previous study participants. Inclusion criteria were: age 18–50 year; BMI 18–30 kg/m^2^; fasting blood HbA1c < 48 mmol/mol (< 6.5%) and not diagnosed with diabetes; fasting total blood cholesterol < 7.8 mmol/l; fasting blood triglycerides < 2.3 mmol/l; normal liver function (assessed via blood liver enzyme measurement); blood pressure < 140/90 mmHg; willingness to refrain from pre- and probiotics, vitamin supplements as well as alcoholic beverages during the study. Exclusion criteria were gastrointestinal disease; use of medications that affect gastrointestinal motility; use of antibiotic, prebiotic, or probiotics in previous 3 months; use of tobacco or recreational drugs and history of coronary artery disease, diabetes, or other chronic disorders. Individuals who had been enrolled in dietary trials in the previous 3 months were also excluded. Participants took part in a screening visit where a fasting blood sample, blood pressure and anthropometric measurements were taken to confirm study eligibility based on the above inclusion criteria.

### Study design and interventions

Mycomeat was an investigator blind, randomised crossover controlled study consisting of 2 × 2-week feeding blocks separated by a 4 week washout, where participants returned to their usual dietary habits [28]. During study phases, participants were provided with 240 g/day (uncooked weight) of either red and processed meat products or the weight equivalent of mycoprotein-based products. The food products were also selected to match for similar energy content. The diets were built around a seven-day rotation of food products, in line with real world exposures. Details of the study diets have been outlined in detail previously [28].

Participants were asked to avoid additional high-protein, fibre, or probiotic supplements, and to refrain from alcohol consumption for the duration of the trial, but to otherwise maintain their usual diet. During each phase of the study, participants were provided and asked to complete a compliance document which outlined the amount of study foods they consumed each day. In addition, participants completed a 1-day food record, from which energy and macronutrient intake was estimated using Nutritics nutrition analysis software (version 5.66 Education) [29].

### Sample collection and anthropometric measurements

At baseline and the conclusion of each intervention period, participants visited the Brain, Performance and Nutrition Research Centre (BPNRC, Northumbria University, Newcastle, UK) in an overnight fasted state. Anthropometric measurements of body weight, body mass index (BMI), hip and waist circumference, systolic blood pressure (SBP), diastolic blood pressure (DBP) and body fat percentage by bioimpedance scale (Tanita BC-418) were taken. Venous blood samples were collected in serum SST and fluoride oxalate tubes for analysis of blood lipids and glucose, and EDTA tubes for the analysis of lipoprotein particle sub fractions (Fisher Scientific). In addition, at each visit, a morning spot urine sample was collected and stored on ice during the study visit before the sample was aliquoted into sterile 1.5-ml tubes and stored at − 80 °C until further analysis.

### Blood biochemical measurements

Blood collected in serum SST and fluoride oxalate tubes was analysed on the same day for fasting serum triglycerides, total cholesterol, and plasma glucose via routine automated clinical biochemistry at the Blood Sciences Department of The Royal Victoria Infirmary Hospital, Newcastle, UK. Blood that was collected in EDTA tubes was kept at 4 °C prior to centrifugation at 1900 g at 4 °C for 10 min, and the plasma collected was stored in aliquots (0.5 ml) at − 80 °C until analysis. Plasma HDL cholesterol was measured by enzymatic endpoint analysis using enzyme reagent kits on a clinical chemistry analyser (Randox Daytona Series). LDL cholesterol was estimated using the Friedewald equation [30].

### LC–MS quantification of TMAO

Urine samples were specific gravity normalised and prepared as previously described [28].

Trimethylamine N-oxide (TMAO) was then quantified by Hydrophilic Liquid Interaction Chromatography (HILIC) in positive mode, performed on a Vanquish liquid chromatography chromatographic separation system connected to an IDX High Resolution Mass Spectrometer (Thermo Scientific). The HILIC positive data set was processed via Compound Discoverer 3.2 according to the following settings: untargeted metabolomic workflow with online database: mass tolerance 10 ppm, maximum shift 0.3 min, alignment model adaptive curve, minimum intensity 500 K, S/N threshold 3, compound consolidation, mass tolerance 10 ppm, RT tolerance 0.3 min. Database matching was performed at MS2 level using Thermo scientific m/z cloud with a similar index of 80% or better. TMAO intensity was then identified in the overall metabolite dataset and log transformed for statistical analysis.

### Urinary sodium and potassium

Gravity normalised urine samples were thawed prior to dilution to 1:100 with distilled water. Sodium and potassium concentrations were determined with a flame photometer (PFP-7, Jenway, UK). Standards of sodium (2–8 ppm) and potassium (5–40 ppm) were used to quantify levels prior to transforming into concentrations. Results are expressed in micromoles.

### Urinary nitrates and nitrites

Urinary nitrates and nitrites were determined using chemiluminescence as described previously [31]. Gravity normalised urine samples were thawed prior to dilution to 1:50 with distilled water. To determine nitrite concentrations, 50 μl of urine was injected into a purge vessel containing 8 ml glacial acetic acid and 2 ml aqueous potassium iodide (50 mg/ml). Nitrogen was bubbled through a glass frit to mix the sample and transfer released nitric oxide to a Sievers NOA 280 analyser (Sievers, Boulder, CO, USA) via a condenser, a NaOH (1 mol/l) trap and a polypropylene filter (0.2 μm; Whatman, USA). The signal was processed using the instrument software. After every 6 injections, the purge vessel was emptied and refilled with fresh reagents. For quantification, known standards of sodium nitrite (1–10,000 nmol) were injected into the purge vessel filled with 8 ml glacial acetic acid and 2 ml aqueous potassium iodide (50 mg/ml).

For nitrate determination, 50 μl of urine was injected into the purge vessel containing 8 ml vanadium (III) chloride solution (~ 0.4 g vanadium (III) chloride in 50 ml 1 M hydrochloric acid). The purge vessel was fitted with a water jacket to allow heating of the reagent to 96 °C and cold-water condenser (6 °C), using a circulating bath. Thereafter, the purge vessel was replenished with reagents as described above. The samples were quantified by comparing the area to the area of known standards of sodium nitrate (1–10,000 nmol). Results are expressed as millimoles and micromoles of nitrates and nitrites respectively.

### Assessment of CVD risk

While our study population was young and metabolically healthy, we derived a panel of CVD risk scores to determine if the study diets would impact putative CVD risk. These included the Framingham Risk Score (FRS), based on age, gender, systolic blood pressure, smoking habit, total cholesterol, HDL cholesterol and presented as % risk of CVD in the next 10 years [32]. The QRESEARCH risk estimator version 3 (QRISK3), based on age, gender, ethnicity, total cholesterol to HDL cholesterol ratio, systolic blood pressure, height, weight, smoking habits, diabetes status, and other clinical information related to medication, treatment and chronic disease [33]. QRISK3 also provides a 10 year % CVD risk estimate. We also applied the atherosclerotic cardiovascular disease (ASCVD) risk score, based on age, gender, ethnicity, total cholesterol, HDL cholesterol, systolic blood pressure, height, smoking habits and diabetes status [34]. The ACSVD provides both a 10 year and lifetime risk score.

### Statistical analysis

This study was powered according to the primary endpoint, change in faecal genotoxicity, described previously [28]. Recruitment was continuous until completion of the study at n = 20.

Data was assessed for normality by visualising Q-Q plots and performing Shapiro-Wilks tests before statistical analysis was performed. Data assessed to be non-normally distributed was analysed  with non-parametric tests. Changes from baseline within study phases and differences between study phases in all measures was assessed using mixed effects models. In all models, age, BMI and habitual alcohol intake were included as fixed effects, with the participant as the random effect. For measures related to BMI (body weight, body fat, trunk fat, and BMI itself), BMI was excluded from the model to avoid collinearity. The interaction of the order at which the participants received the diets was also included to capture the impact of diet order on study outcomes.

Gut microbiome data for Mycomeat has been reported in detail elsewhere [28] and supporting meta data is available online (https://doi.org/10.25398/rd.northumbria.c.6010252). Here, in an exploratory sub analysis, we investigated whether the gut microbiota at baseline was associated with the influence of the study diets on LDL cholesterol. To address this question, orthogonal projections to latent structure (OPLS) was constructed on the abundance of microbial genera at baseline of responders (participants whose blood LDL was lower and/or reduced greater following the mycoprotein phase compared to the meat phase) and non-responders (participants whose blood LDL was lower and/or reduced greater after the meat phase compared to the mycoprotein phase). We then applied random forest modelling [35] to identify any microbial phyla which could discriminate responders from non-responders at baseline.

A P value of ≤ 0.05 was considered significant. All statistical analyses and visualisations were performed in RStudio [36].

## Results

### Participants

Twenty participants completed the study, the mean age at baseline was 30.4 years, and the average BMI was 24. Participant enrolment began on 1 June 2019 and the date of final data collection was 29 January 2020. Participant baseline characteristics are reported in Table 1 and the study CONSORT diagram can be found elsewhere [28].

**Table 1:** Participants’ baseline characteristics (*n* = 20)

Age, years	30.4 ± 7.92
Anthropometric parameters	
Weight, kg	80.6 ± 10.90
BMI, kg/m^2^	24.0 ± 2.87
Waist circumference, cm	86.9 ± 8.16
Body fat, %	15.5 ± 5.56
Trunk fat, %	16.3 ± 6.90
Systolic blood pressure, mmHg	126 ± 12.2
Diastolic blood pressure, mmHg	71.5 ± 9.27
Blood parameters	
Total cholesterol, mmol/l	4.32 ± 0.83
HDL Cholesterol, mmol/l	1.57 ± 0.43
LDL Cholesterol, mmol/l	2.29 ± 0.88
Triglycerides, mmol/l	0.89 ± 0.46
Glucose, mmol/l	4.86 ± 0.45
Dietary intake	
Energy, kcal/day	2515.59 ± 754.85
Protein, g/day	126.44 ± 45.42
Protein, % of energy	20.10 ± 7.22
Carbohydrate, g/day	281.20 ± 81.41
Carbohydrate, % of energy	44.71 ± 12.94
Fat, g/day	98.37 ± 46.75
Fat, % of energy	35.19 ± 16.73
Saturated fat, g/day	35.94 ± 21.22
Saturated fat, % of energy	12.86 ± 7.59
Fibre, g/day	26.16 ± 7.79
Sodium, mg/day	2853.02 ± 1243.93
Total meat intake, g/day	221.60 ± 176.87
Red and processed meat intake g/day	80.25 ± 83.47
Mycoprotein intake, g/day	0 ± 0

Data are presented as means ± SDs

### Effect of intervention on nutritional intake

Overall, there was a good level of compliance to the intervention, as well as no adverse reactions or symptoms reported by participants. Self-reported fibre intake was significantly higher during the mycoprotein phase compared to the meat phase (+ 16.74 ± 2.92 g/day, *P* < 0.001). Total energy (+ 212.26 ± 242.63 kcal/day, *P* = 0.47), carbohydrate (+ 4.07 ± 2.32% energy, *P* = 0.07) and sodium (+ 514.87 ± 424.95 mg/day, *P* = 0.32) were also higher during the mycoprotein phase, whereas protein (+ 2.07 ± 1.28% energy,* P* = 0.13), fat (+ 2.74 ± 2.65% energy, *P* = 0.27) and saturated fat (+ 1.46 ± 1.32% energy, *P* = 0.21) were higher during the meat phase. Apart from the difference in fibre, there were no significant differences between study phases (Table 2).

**Table 2:** Difference in self-reported nutritional intake between diets, including provided study food

Nutrient	Meat	Mycoprotein	Difference^a^	*P*^b^
Energy, kcal/day	2355.18 ± 553.32	2567.44 ± 796.97	+ 212.26 ± 242.63	0.47
Protein, % of energy	20.28 ± 3.85	18.21 ± 3.38	− 2.07 ± 1.28	0.13
Carbohydrate, % of energy	42.23 ± 6.24	46.30 ± 6.85	+ 4.07 ± 2.32	0.07
Fat, % of energy	37.27 ± 7.31	34.53 ± 7.70	− 2.74 ± 2.65	0.27
Saturated fat, % of energy	13.95 ± 3.78	12.49 ± 3.81	− 1.46 ± 1.32	0.21
Fibre, g/day	26.52 ± 8.40	43.26 ± 8.69	+ 16.74 ± 2.92	< 0.001
Sodium, mg/day	2714.25 ± 979.59	3229.12 ± 1405.28	+ 514.87 ± 424.95	0.32

Values are presented as means ± SDs. Nutrient intake calculated from 1 day food records using Nutritics nutritional analysis software

^a^Differences between nutrient intake during Mycoprotein compared to Meat presented as least square means ± SEs

^b^*P* values were calculated for differences between study phases using mixed effects models. A *P* ˂ 0.05 was considered significant

### Blood biochemistry

Following the mycoprotein diet, both total cholesterol (-0.33 ± 0.13 mmol/l, *P* = 0.02) and LDL cholesterol (− 0.32 ± 0.10 mmol/l, *P* = 0.005) were significantly reduced from baseline, with the difference between the study phase effects also significant (difference total cholesterol, 0.37 ± 0.14 mmol/l, *P* = 0.02: difference LDL, 0.34 ± 0.13 mmol/l,* P* = 0.01). There was no statistically significant difference in triglycerides between study phases (+ 0.19 ± 0.11 mmol/L, *P* = 0.09 for mycoprotein relative to meat). HDL cholesterol and plasma glucose were not significantly affected by the intervention (Table 3).

**Table 3:** Effects of 2 week diet phases on anthropometric, blood and urinary markers

Variable	Δ Meat	Δ Meat (%)	*P*^b^	Δ Mycoprotein	Δ Mycoprotein (%)	*P*^b^	Difference	*P*^b^
Anthropometric parameters^a^								
Weight, kg	+ 0.26 ± 0.22	+ 0.28	0.33	− 0.17 ± 0.27	− 0.18	0.58	− 0.43 ± 0.33	0.23
BMI, kg/m^a^	+ 0.09 ± 0.06	+ 0.34	0.22	− 0.04 ± 0.08	− 0.15	0.63	− 0.13 ± 0.10	0.24
Body fat, %	+ 0.33 ± 0.25	+ 1.96	0.42	+ 0.18 ± 0.39	+ 0.66	0.82	− 0.15 ± 0.48	0.76
Trunk fat, %	+ 0.62 ± 0.39	+ 5.77	0.09	+ 0.41 ± 0.51	+ 1.63	0.82	− 0.21 ± 0.65	0.76
Waist circumference, cm	+ 0.80 ± 0.39	+ 0.92	0.05	− 0.15 ± 0.31	− 0.15	0.67	− 0.95 ± 0.42	0.04†
Systolic blood pressure, mmHg	+ 1.70 ± 1.40	+ 1.47	0.27	− 2.41 ± 1.89	− 1.65	0.23	− 4.11 ± 2.47	0.11
Diastolic blood pressure, mmHg	+ 1.77 ± 1.23	+ 2.53	0.23	− 0.80 ± 1.23	− 1.17	0.43	− 2.57 ± 1.73	0.16
Blood parameters								
Total cholesterol, mmol/l	+ 0.04 ± 0.09	+ 1.34	0.66	− 0.33 ± 0.13	− 6.74	0.02*	− 0.37 ± 0.14	0.02†
HDL Cholesterol, mmol/l	+ 0.05 ± 0.05	+ 4.67	0.34	− 0.04 ± 0.05	− 1.52	0.42	− 0.09 ± 0.07	0.21
LDL Cholesterol, mmol/l	+ 0.02 ± 0.08	+ 1.43	0.95	− 0.32 ± 0.10	− 12.30	0.005*	− 0.34 ± 0.13	0.01†
Triglycerides, mmol/l	− 0.06 ± 0.08	− 0.32	0.42	+ 0.13 ± 0.09	+ 28.89	0.09	+ 0.19 ± 0.11	0.09
Glucose, mmol/l	+ 0.13 ± 0.13	+ 4.01	0.29	+ 0.15 ± 0.14	+ 3.23	0.48	+ 0.02 ± 0.20	0.92
Urinary parameters								
TMAO, log intensity	-0.06 ± 0.05	− 0.76	0.50	+ 0.08 ± 0.04	+ 1.16	0.09	+ 0.14 ± 0.07	0.06
Sodium, μmol	+ 10.30 ± 25.50	+ 19.66	0.58	+ 27.25 ± 22.10	+ 14.34	0.25	+ 16.95 ± 11.80	0.71
Potassium, μmol	− 168.38 ± 58.60	− 24.10	0.006*	− 42.26 ± 41.30	− 2.45	0.32	+ 126.12 ± 50.30	0.02†
Nitrates, mmol	− 1.72 ± 0.68	− 13.23	0.04	+ 0.39 ± 0.60	+ 25.88	0.44	+ 2.12 ± 0.90	0.04†
Nitrites, μmol	− 0.77 ± 0.47	− 2.54	0.35	− 0.37 ± 0.39	− 1.20	0.29	+ 0.40 ± 0.63	0.97

Differences between variables at the beginning and end of each diet are shown along with % change. The difference column shows the differences between variables at the end of the mycoprotein phase compared with the end of the meat phase. *P* values were calculated for changes within and differences between study phases using mixed effects models. Values are presented as least square means ± SEs for changes within and differences between study phases. % change are presented as least square means

^a^For measures related to BMI (body weight, body fat, trunk fat, and BMI itself), BMI was excluded from the model to avoid collinearity

^b^A *P* < 0.05 was considered significant.*Mean change significantly different from baseline.† Mean change significantly different between Mycoprotein and Meat dietary periods

### Blood pressure

There was no statistically significant change from baseline in SBP  or DBP  after the meat phase (SBP, + 1.70 ± 1.40 mmHg; *P* = 0.27, DBP, + 1.77 ± 1.23 mmHg; *P* = 0.23), nor were there statistically significant changes from baseline following the mycoprotein phase (SBP, − 2.41 ± 1.89 mmHg; *P* = 0.23, DBP, − 0.80 ± 1.23 mmHg; *P* = 0.43). The difference between study phases (SBP − 4.11 ± 2.47 mmHg DBP − 2.57 ± 1.73 mmHg mycoprotein relative to meat) was also not statistically significant (SBP *P* = 0.11, DBP *P* = 0.16) (Table 3).

### Anthropometry

Over the course of the study, there were no significant effects on body weight (meat, + 0.26 ± 0.22 kg; *P* = 0.33; mycoprotein, − 0.17 ± 0.27 kg; *P* = 0.58), BMI (meat, + 0.09 ± 0.06 kg/m^2^; *P* = 0.22; mycoprotein, − 0.04 ± 0.08 kg/m^2^; *P* = 0.63), or body fat (meat, + 0.33 ± 0.25%; *P* = 0.42; mycoprotein, + 0.18 ± 0.39%; *P* = 0.82). Following the meat phase, waist circumference increased (+ 0.80 ± 0.39 cm) while there was a reduction after the mycoprotein phase (− 0.15 ± 0.31 cm). Following mycoprotein, waist circumference was 0.95 cm lower than after the meat phase (*P* = 0.04) (Table 3).

### Urinary markers

Urinary excretion of sodium was not significantly different from baseline following either arm of the intervention (meat, *P* = 0.58; mycoprotein, *P* = 0.25), the difference between study phases was also not significant (*P* = 0.71). Potassium excretion did not differ markedly from baseline after the mycoprotein phase (*P* = 0.32) but was significantly reduced following the meat phase (*P* = 0.007), the difference between study phases was also significant (*P* = 0.02) (Table 3).

Urinary nitrates were significantly reduced following the meat phase (− 1.72 ± 0.68 mmol, *P* = 0.04),  but were unaffected  following the mycoprotein phase (+ 0.39 ± 0.60 mmol*, P* = 0.44), the difference between study phases was also significant (2.12 ± 0.90 mmol*, P* = 0.04). The diets had negligible effects on the excretion of urinary nitrites, with a small reduction from baseline values following both (Table 3). Urinary TMAO excretion was not significantly affected within or between study phases (Table 3).

### CVD risk assessment

There were no statistically significant effects on the panel of CVD risk scores. As expected, the derived scores were low for the participants at all time points. The mycoprotein phase led to marginal reductions across the risk scores, whereas the risk scores were either unaffected or increased following the meat phase (Table 4).

**Table 4:** Effects of 2 week diet phases on CVD risk

	Mycoprotein baseline	Mycoprotein completion	Change	*P*^d^	Meat baseline	Meat completion	Change	*P*^d^	Difference in diet effects^c^	*P*^d^
FRS^a^	2.03	1.74	− 0.29	0.19	1.86	1.91	+ 0.05	0.99	− 0.34	0.24
QRISK3^b^	0.58	0.50	− 0.08	0.35	0.54	0.54	+ 0.00	0.77	− 0.08	0.44
ASCVD 10 year^c^	0.78	0.68	− 0.10	0.27	0.78	0.78	+ 0.00	0.46	− 0.10	0.51
ASCVD lifetime	31.80	31.30	− 0.50	0.68	31.80	33.85	+ 2.05	0.99	− 2.55	0.72

Results presented as % CVD risk. The difference in diet effects column shows the Mycoprotein phase effects compared with at the Meat phase effects. *P* values were calculated using mixed effects models

^a^Framingham Risk Score (FRS) based on the following risk factors, age, gender, systolic blood pressure, smoking habit, total cholesterol and HDL cholesterol

^b^QRISK3 score based on following risk factors, age, gender, ethnicity, total cholesterol to HDL cholesterol ratio, systolic blood pressure, height, weight, smoking status, diabetes status, and other clinical information related to medication, treatment and chronic disease

^c^Atherosclerotic cardiovascular disease (ASCVD) 10 year and lifetime risk score based on following risk factors, age, gender, ethnicity, total cholesterol, HDL cholesterol, systolic blood pressure, height, smoking status and diabetes status

^d^A *P* < 0.05 was considered significant

## Discussion

Here, we report the effects of replacing red and processed meat with the weight equivalent of mycoprotein based foods over two weeks, on markers of CVD risk in metabolically healthy male adults. We note that these observations are of a priori defined secondary endpoints in a study designed to evaluate measures of gut health, thus we caution the potential for type 1 error. Nevertheless, our observation that mycoprotein consumption elicits reductions in total (− 6.74%) and LDL cholesterol (− 12.3%) in metabolically healthy and relatively young British men are of value; these findings reinforce similar observations from a limited number of other relatively small intervention trials in different study populations. Notably, in two parallel feeding studies, using mycoprotein based test foods purposefully designed for the intervention, Turnbull et al. noted that, (1) 191 g daily mycoprotein for 3 weeks reduced both total (− 13%) and LDL cholesterol (− 9%) in 9 subjects with slightly elevated baseline cholesterol status [26]; and (2) that 130 g/day over 8 weeks reduced total (− 8%) and LDL cholesterol (− 13%) in a similar study population (n = 11) [25]. Using commercially available products, Ruxton and McMillan [37] recorded no change in total and LDL cholesterol in 21 British adults consuming, by product preference, the equivalent of 88 g mycoprotein daily, however this was a lightly controlled open label trial in a free-living population, and they did note evidence of an effect with higher levels of compliance, and a reduction in cholesterol in those with higher baseline values. More recently, Coelho et al. [24] reported a reduction in total (− 14.3%) and LDL (− 19.33%) cholesterol in metabolically healthy young adults (n = 20) consuming the equivalent of 181 g wet weight mycoprotein per day for 1 week. The magnitude of change in LDL (− 12.3%) that we observed with mycoprotein exceeds that achievable through consuming 2.4 g/day of plant sterols and stanols, which is deemed clinically meaningful by the European Food Standards Agency [38]. However, probably due to the nature of our healthy study population (with baseline total cholesterol < 5 mmol/l), the intervention did not have a significant effect on a panel of CVD risk scores [39], and we recommend that future studies target higher risk groups.

Overall, there is now consistent evidence across several, albeit small, intervention studies demonstrating that displacement of meat with mycoprotein decreases cholesterol [40, 41]. Whether or not mycoprotein is uniquely cholesterol lowering in the context of a high-fibre low-meat diet is uncertain, for example, SWAP-MEAT was an 8-week randomised controlled trial with no washout in which healthy volunteers consumed ≥ 2 portions per day of a variety of meat or meat substitutes, none of which were mycoprotein based [42]. In that study, volunteers on the meat substitute arm reported consuming an additional ~ 6 g of fibre per day and showed a difference of 0.27 mmol/l LDL between study arms. In Mycomeat, our volunteers consumed a significantly higher amount of total fibre (+ 16.74 g/day) on the mycoprotein arm, leading to a greater net reduction in LDL compared to the meat arm (0.34 mmol/l) than the reduction observed in SWAP-MEAT. A study comparing meat alternatives with different protein constituents, whilst controlling total fibre intake would further the understanding about their unique beneficial properties.

Whilst a definitive intervention study demonstrating that mycoprotein, independently of meat displacement, lowers cholesterol is still needed, there are several mechanisms that might explain the observed effect. (1) In a previous health claim assessment, an EFSA panel concluded that the hypocholesteroleamic properties of mycoprotein were simply a reflection of its *β*-glucan content [43]. We do note however that the *β*-glucan (*β*1-3, *β*1-6) in mycoprotein has a different bonding arrangement to *β*-glucan found in oats and barley (*β*1-3, *β*1-4). It also has different sugar chain lengths and a different degree of branching which affect its viscosity [44, 45]. The hypocholesterolaemic effects of oat *β*-glucan is considered a function of its viscosity, and subsequent ability to bind bile and cholesterol. In support of such a mechanism for mycoprotein, Colosimo et al. [46] observed that mycoprotein may both inhibit intestinal lipases and sequester bile in an in vitro model. However, the authors argue that the bile sequester is a function of the fungal hyphae and the food matrix as opposed to its *β*-glucan content. Notably, we were not able to identify any increase in faecal cholestenone or bile acids in our metabolomic analysis that would suggest increased cholesterol clearance (Supplementary material, Fig. S1). (2) The intestinal microbiota also influences enterohepatic circulation, through its bile and cholesterol hydrolase activity [47]. We have previously described the effects of this intervention on the composition of the microbiome where we did note an increase in the abundance of *Lactobacilli* which are known to be bile and cholesterol hydrolase capable [47, 48]. Further, in support of this potential explanation, in an exploratory sub-analysis of the microbiome data, we noted differences in baseline microbiome between those we classified as ‘LDL responders’ versus ‘non-responders’ to the mycoprotein diet (Supplementary material, Figs. S2–4). (3) Microbially produced short chain fatty acids (SCFA) influence endogenous cholesterol synthesis [49]. The principal SCFA, acetate, is a substrate for both lipid and cholesterol synthesis, however, its incorporation into cholesterol may be inhibited by the presence of propionate [50], and the overall balance of acetate relative to propionate has been suggested as a predictor of cholesterol status [51]. Propionate and butyrate are also putative inhibitors of hepatic cholesterol synthesis through disruption of sterol regulatory element-binding protein (SREBP) signalling and down regulation of key enzymes of the β-hydroxy β-methylglutaryl-CoA (HMG-CoA) pathway [49, 52, 53]. In support of this potential mechanism, we have previously described the effect of this intervention on SCFA excretion and note higher propionate production with mycoprotein. Thus, future studies should seek to explore these potential mechanisms in respect to mycoprotein and cholesterol status.

TMAO is considered a marker of CVD risk [54] and has been selected as the primary endpoint in recent randomised controlled trials, including SWAP-MEAT [14, 42]. We did not however observe statistically meaningful differences in TMAO in this intervention. Future work could consider a dedicated study design with a primary focus on mycoprotein consumption and TMAO production.

Thus far, three acute feeding studies have reported on the effect of consuming mycoprotein on glycaemic response in healthy volunteers. In particular, Turnbull et al. [55] noted that 75 g of mycoprotein suppresses glycaemic response following a carbohydrate challenge relative to a protein matched soya-based control in healthy volunteers. Bottin et al. [56] observed no influence on post-test-meal glucose but a lower insulinemic response to mycoprotein relative to an iso-energetically matched serving of chicken in overweight but otherwise metabolically healthy volunteers. Most recently, Dunlop et al. [57] demonstrated a more controlled insulinemic response to a mass matched serving of liquefied mycoprotein relative to milk. To our knowledge, only one prior study has considered insulin sensitivity and glucose control over a longer duration. In that study by Coelho et al. [24], 2 servings a day of mycoprotein had no effect on glycaemia after a 1 week intervention. These null observations mirror our own in relation to fasted glucose, however we caution that both studies were carried out in metabolically healthy volunteers, with glucose being measured as secondary endpoints. Mechanistically, Colosimo et al. [58] showed that the cell wall fraction of *Fusarium venanatum* may entrap α-amylase and thereby reduce starch hydrolysis. Supplementary oat *β*-glucan consistently lowers the glucose and insulin response to high carbohydrate meals [59], and National Diet and Nutrition Survey data suggest that regular consumers of mycoprotein have lower glycated HbA1c [60]. In the context of metabolic health, we also noted, in our 2-week high-dose intervention, a small but statistically significant difference in waist circumference. This may be consistent with a handful of acute feeding studies noting increased satiation and desire to eat relative to control [61, 62]. However longer interventions in higher metabolic risk groups are needed to fully determine any potential influence of mycoprotein in adiposity and metabolic health.

We observed non-statistically significant, but potentially clinically meaningful trends towards reduced systolic and diastolic blood pressure with mycoprotein (− 4.11 mmHg and − 2.71 mmHg relative to the meat diet). The effects of mycoprotein on blood pressure has not been reported previously. However, a reduction in blood pressure may be consistent with a diet high in fibre, alongside the small observed reduction in waist circumference, and greater excretion of potassium and urinary nitrates relative to the meat arm.

Strengths of this work include the use of a randomised controlled crossover study design, whereby the participants acted as their own control. A parallel design would have required much larger numbers and matching of characteristics to produce equivalent statistical robustness. The investigators were also blinded to the intervention, removing any chance of bias from the analysis. The mycoprotein and meat products included were not exclusively produced for the study and are readily available and consumed amongst the population, The intervention was based on a high but not unrealistic intake of meat amongst the target group, healthy young UK based men, thus the findings may be translatable and of particular relevance to those consuming high meat diets.

Limitations of this work include that this was an analysis of a priori defined secondary endpoints in a study designed to assess gut health. Further, our study population was relatively young and comprised only healthy, male adults. Compliance was only assessed using a 1-day food record, this was primarily due to concerns over participant burden and was balanced against the fact that nutritional status was not a primary outcome, a more thorough interrogation of compliance might have helped explain why some volunteers responded better than others to the intervention.

In conclusion, this work is timely in the context of the explosion of interest in meat substitutes and the push towards meat reduction for ecological and health purposes. It demonstrates in a real-world setting that substituting mycoprotein for meat improves biochemical markers of cardiovascular disease risk. Further work is needed to evaluate dose response as part of normal dietary patterns and to elucidate mechanisms behind the observed responses.

### Supplementary Information

Below is the link to the electronic supplementary material.

Supplementary file1 (DOCX 729 kb)

## Data Availability

Data described in the manuscript will be made publicly and freely available without restriction at 10.25398/rd.northumbria.c.6010252.
