# Supplementary material for: The effects of substituting red and processed meat for mycoprotein on biomarkers of cardiovascular risk in healthy volunteers: an analysis of secondary endpoints from Mycomeat
Source: Eur J Nutr. 2023 Aug 25;62(8):3349–59. doi: 10.1007/s00394-023-03238-1 (PMC10611638; doi:10.1007/s00394-023-03238-1)

**SUPPLEMENTARY MATERIAL**

**The effects of substituting red and processed meat for mycoprotein on biomarkers of cardiovascular risk in healthy volunteers: an analysis of secondary endpoints from Mycomeat**

**Figure S1.** Cholesterol related metabolites in stool pre and post study phases.

**Figure S2.** Orthogonal projections to latent structures (OPLS) plot of baseline microbial genera discriminating between responders and non-responders in LDL cholesterol response to mycoprotein consumption.

**Figure S3.** Microbial genera with highest loadings in OPLS model to discriminate between responders and non-responders in LDL cholesterol response to mycoprotein consumption.

**Figure S4.** Box plots showing difference in baseline abundance of microbial phylum identified as discriminatory from random forest modelling between responders and non-responders in LDL cholesterol response to mycoprotein consumption.

**Figure 1. Cholesterol metabolite excretion in stool pre and post study phases.**

Nomenclature presented as reported via the LC-MS platform. **A)** Cholest-4-en-3-one **B)** 27-Norcholestane-3,7,12,24,25,26-hexol **C)** 3-aza-?-homocholest-4?-en-4-one **D)** 3beta-hydroxy-4beta-methyl-5alpha-cholest-7-ene-4alpha-carboxylic acid. Error bars represent standard deviation. Changes within study phases and differences between study phases assessed using mixed effects models (P ˂0.05 considered significant).


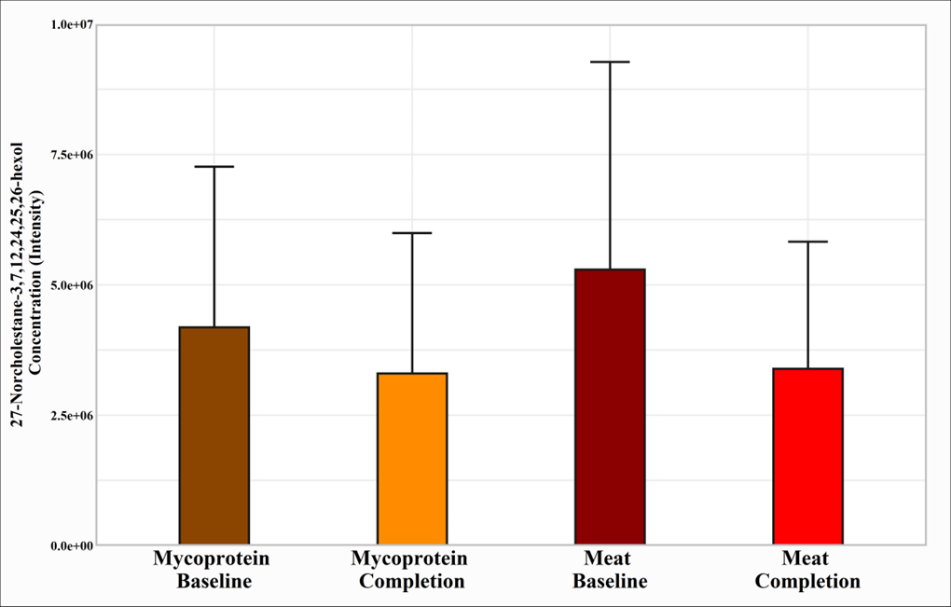

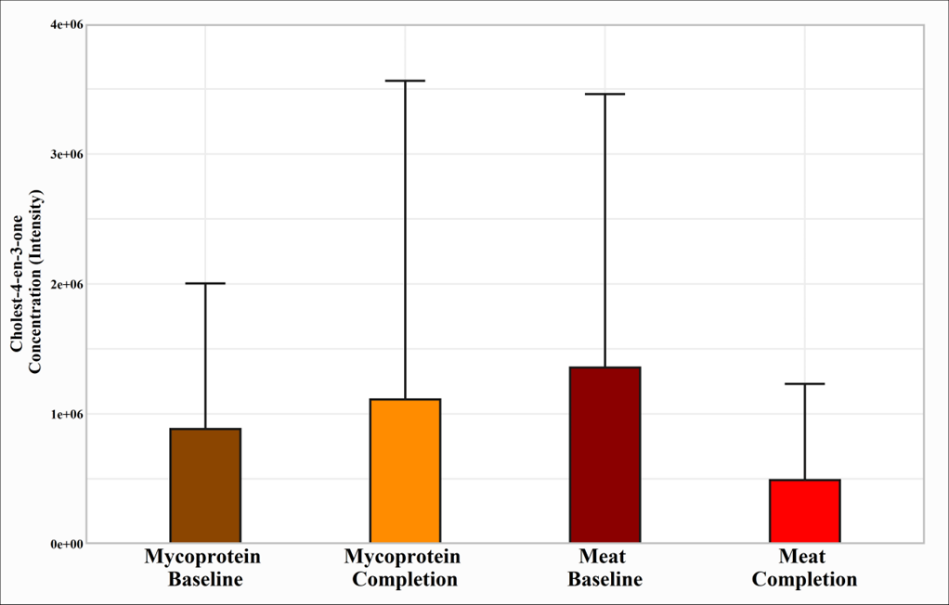

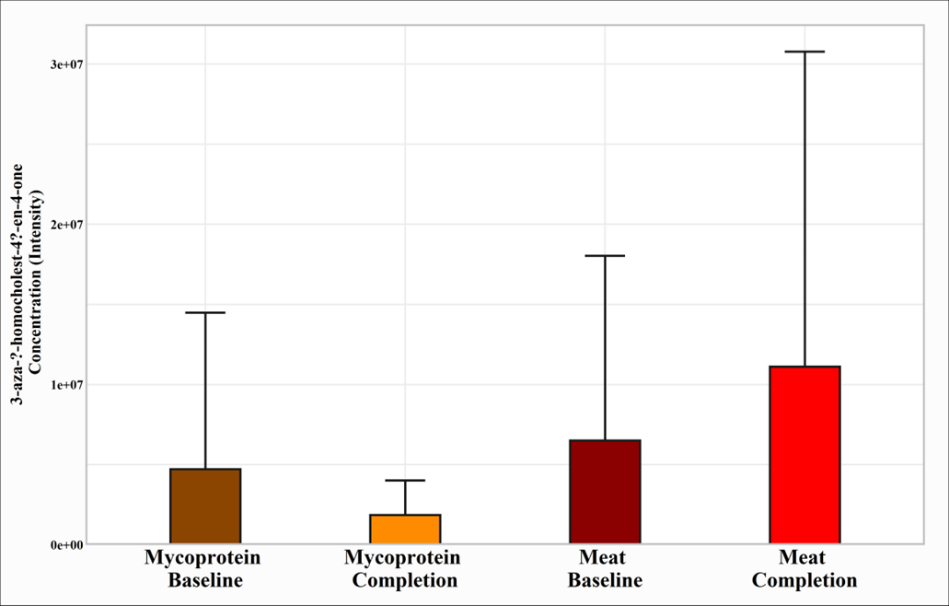

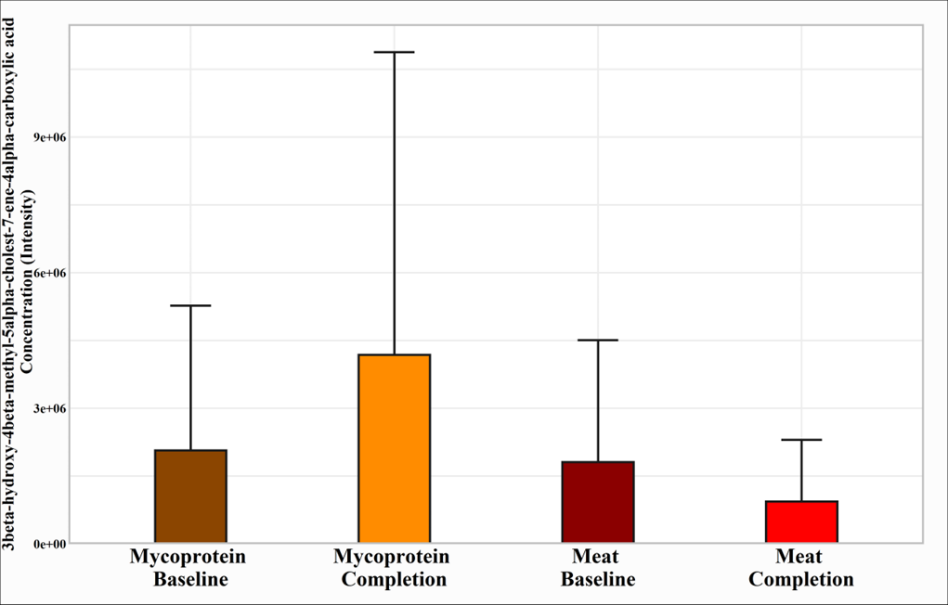


b

d

c

a

**Figure 2. Orthogonal projections to latent structures (OPLS) plot of baseline microbial genera discriminating between responders and non-responders in LDL cholesterol response to mycoprotein consumption.**


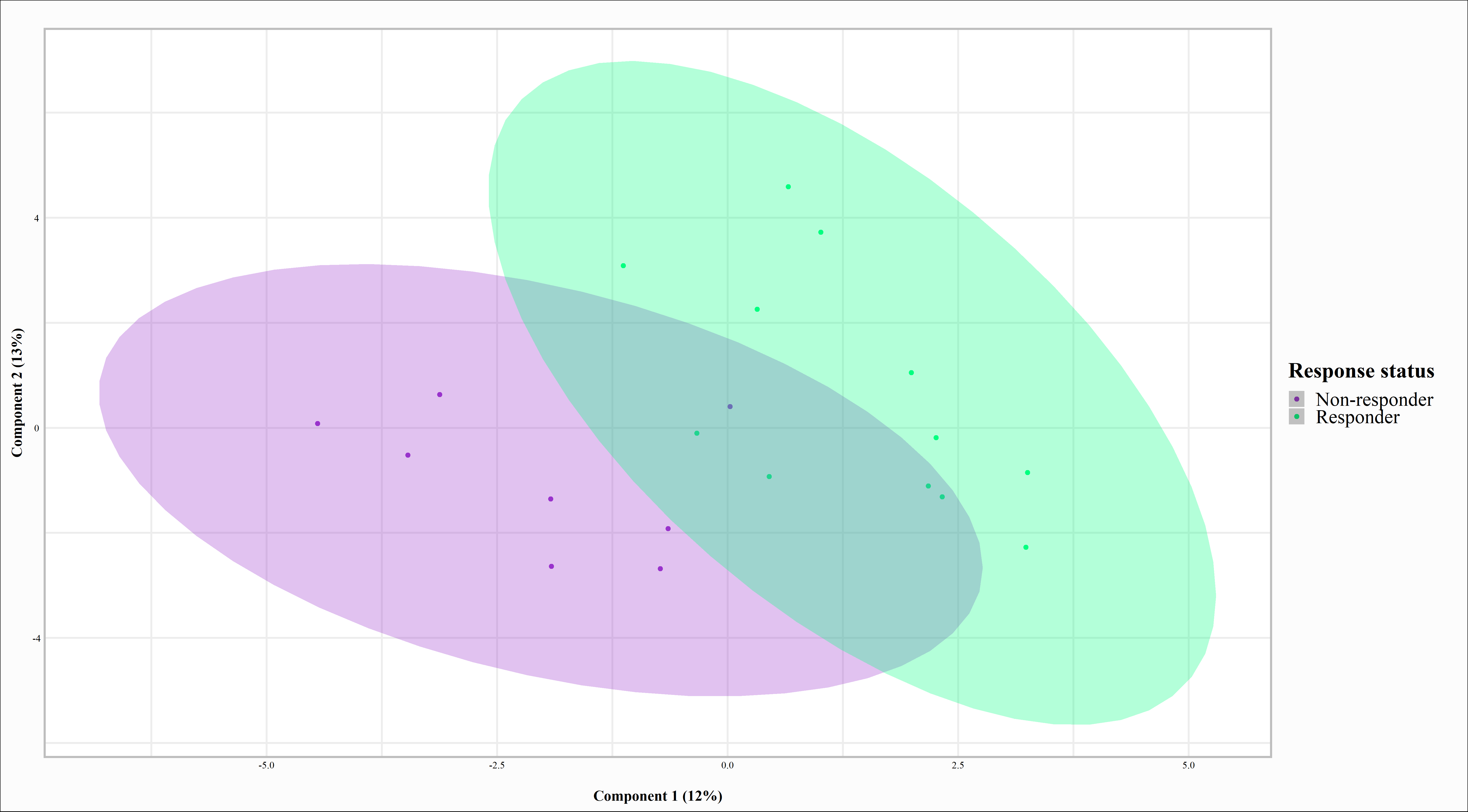


**Figure 3. Microbial genera with highest loadings in OPLS model to discriminate between responders and non-responders in LDL cholesterol response to mycoprotein consumption.**


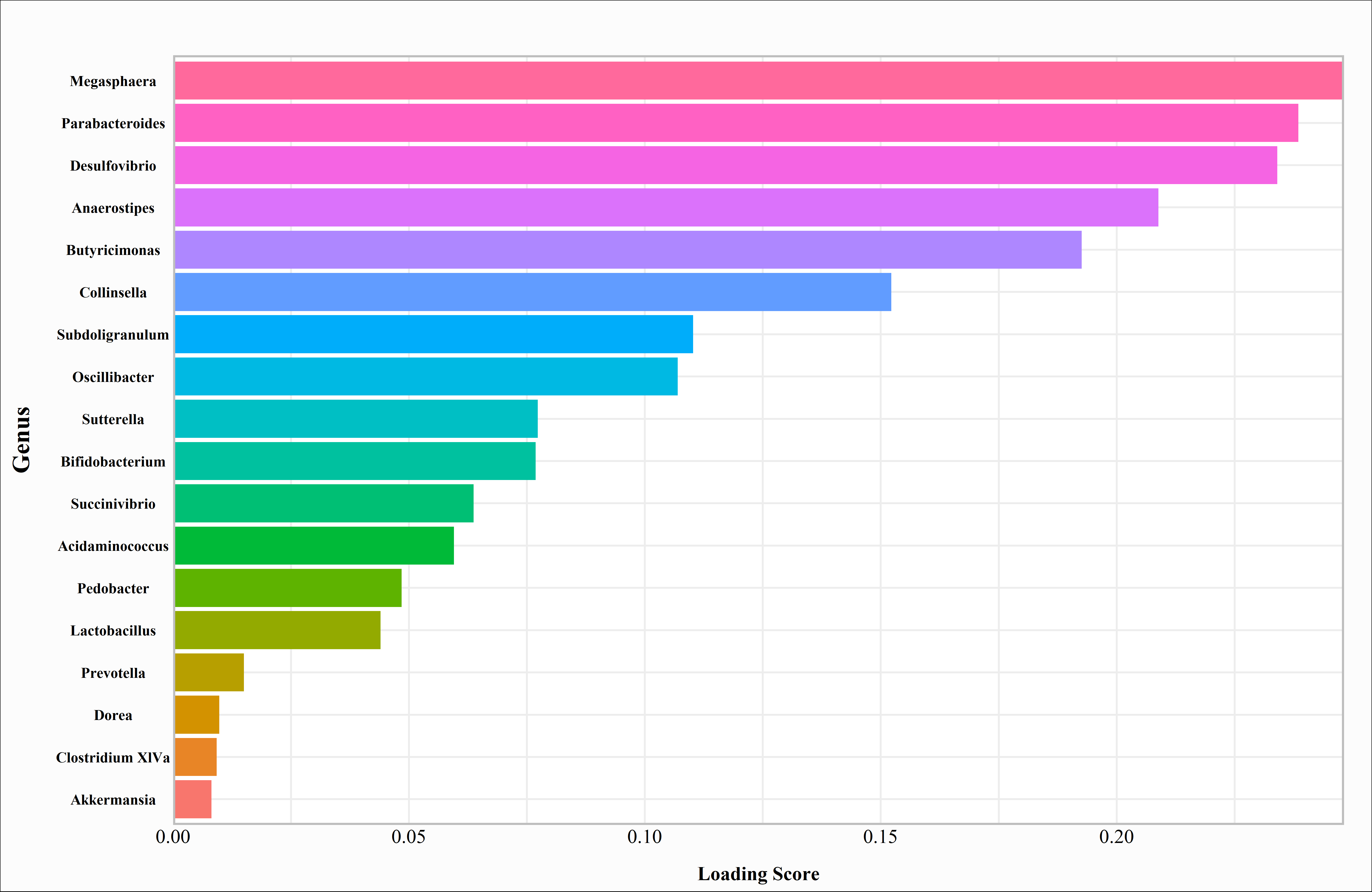


**Figure 4. Box plots showing difference in baseline abundance of microbial phylum identified as discriminatory from random forest modelling between responders and non-responders in LDL cholesterol response to mycoprotein consumption.**


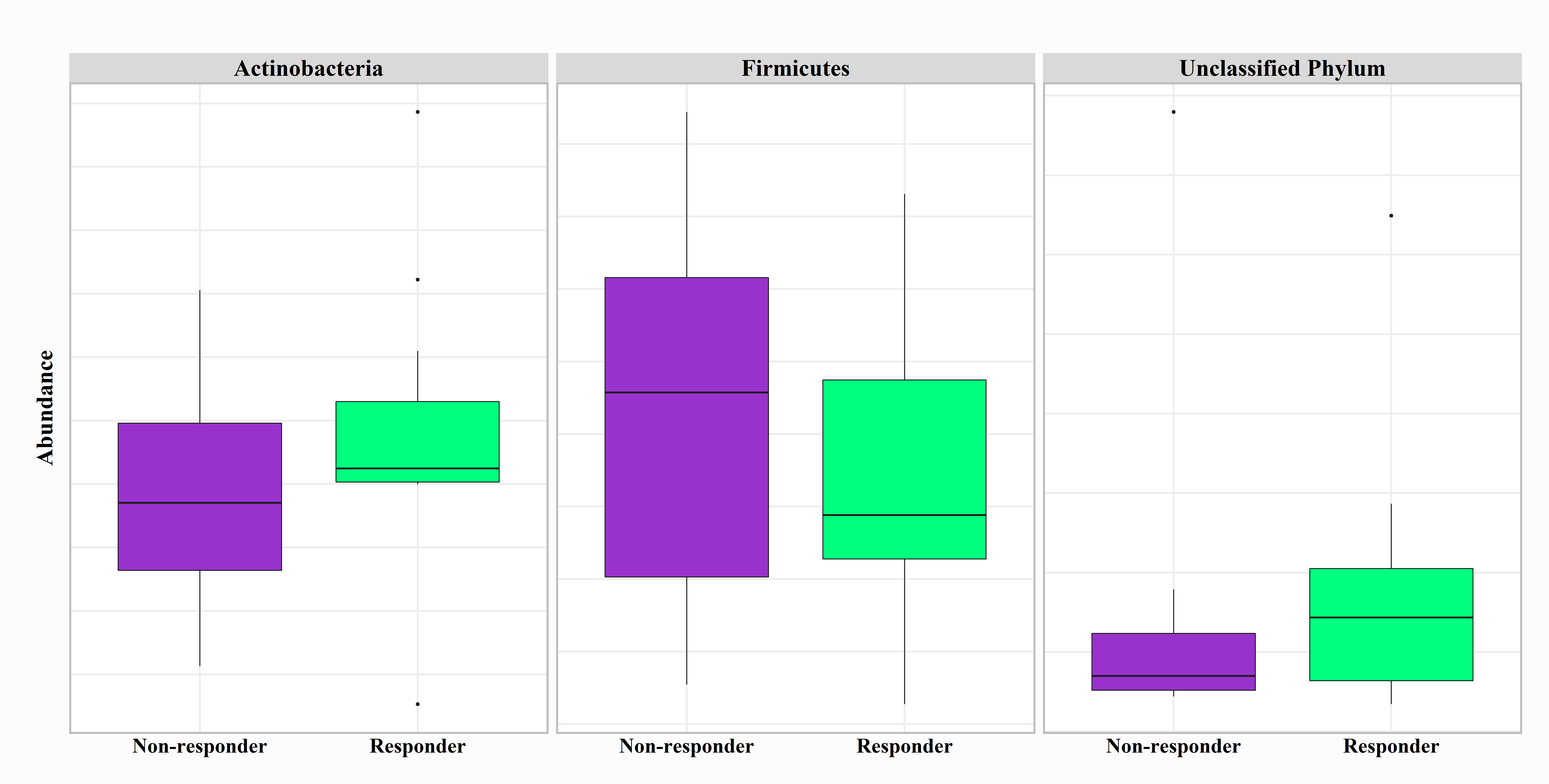

Supplement: Supplementary file 1 — Supplementary file1 (DOCX 729 kb) [file 394_2023_3238_MOESM1_ESM.docx]
